# Biotic therapies for irritable bowel syndrome: current interventions

**DOI:** 10.1093/gastro/goag062

**Published:** 2026-07-13

**Authors:** Gina Wodarczyk, Maria Winte, Diana Mastellone, Termen Singh, Joshua DeSipio, Sangita Phadtare

**Affiliations:** Department of Internal Medicine, Rutgers Robert Wood Johnson Medical School, New Brunswick, NJ 08901, United States; Department of Gastroenterology, Thomas Jefferson University Hospital, Philadelphia, PA 19107, United States; Department of Biomedical Sciences, Cooper Medical School of Rowan University, Camden, NJ 08103, United States; Department of Biomedical Sciences, Cooper Medical School of Rowan University, Camden, NJ 08103, United States; Department of Biomedical Sciences, Cooper Medical School of Rowan University, Camden, NJ 08103, United States; Department of Gastroenterology, Cooper University Hospital, Camden, NJ 08103, United States; Department of Biomedical Sciences, Cooper Medical School of Rowan University, Camden, NJ 08103, United States

**Keywords:** irritable bowel syndrome, gut–brain–microbiome axis, *Bacillus*, *Lactobacillus*, *Bifidobacterium*

## Abstract

Irritable bowel syndrome (IBS) is a common functional gastrointestinal disorder characterized by recurrent abdominal pain and changes in bowel habits without measurable disease processes. Recent research emphasizes the gut–brain–microbiome (GBM) axis and explores how microbiota influence gastrointestinal and central nervous system functions. Here, we first discuss a brief overview of various treatment approaches for IBS, focusing on interventions such as probiotics, prebiotics, or synbiotics that target the GBM axis in adults with IBS. Relevant trials discussed the use of probiotics, prebiotics, or synbiotics. *Bacillus*, *Lactobacillus*, and *Bifidobacterium* featured prominently among the probiotics used. Several significant outcomes, including a reduction in symptom frequency, abdominal-pain severity, and improved stool consistency, were noted. Three prebiotic trials showed variable benefits, including a reduction in bloating and stool consistency, but showed less significant benefits compared with those with probiotics and synbiotics. Six trials on synbiotics showed a significant reduction in abdominal-pain severity, bowel-habit satisfaction, and reduced gut-related anxiety. Probiotics and synbiotics, particularly those containing *Bifidobacteria* or *Lactobacilli* strains, were effective in managing IBS symptoms. Future research is needed to assess their long-term benefits.

## Introduction

Functional gastrointestinal disorders (FGIDs) involve patients perceiving and reporting their illness experiences. In contrast to organic gastrointestinal disorders, which are classified in terms of macro- or micro-level pathology found by physicians, FGIDs are classified in terms of symptoms described by the patients. FGIDs are often perceived as less legitimate than organic disorders, as the former do not require evidence of pathology. To reduce stigmatization, Rome IV redefined FGIDs as disorders of gut–brain interactions (DGBIs), which are characterized by gastrointestinal symptoms related to various combinations of motility disturbance, visceral hypersensitivity, altered mucosal and immune function, altered gut microbiome, and altered central nervous system (CNS) processing [1].

DGBIs are the most common gastrointestinal disorders. Of the 33 adult and 20 pediatric DGBIs, irritable bowel syndrome (IBS) is the prototypical disorder [1]. The published prevalence rates for IBS are broad due to variations in the study populations, data-collection methods, and diagnostic criteria; however, some studies state that it can affect ≤10%–14% of healthy individuals at any given time and is typically characterized by a chronic and fluctuating course [2, 3]. IBS also has a higher prevalence in females compared with males [3]. Sperber *et al*. computed these prevalence rates by using the Rome IV diagnostic criteria for IBS [3]. The Rome IV diagnostic criteria require recurrent abdominal pain occurring weekly for 3 months along with two or more of the following descriptors: related to defecation, associated with a change in stool frequency, or associated with a change in stool form [4]. The classification of IBS depends on the predominant abnormal bowel habit according to the Bristol Stool Form Scale [4]. The four subtypes are IBS with predominant constipation (IBS-C), IBS with predominant diarrhea (IBS-D), IBS with mixed bowel habits (IBS-M), and unclassified IBS (IBS-U) [3, 4].

The American College of Gastroenterology (ACG) provides clinical practice guidelines for the management of IBS. Even when following these guidelines, physicians face challenges in providing treatments that relieve IBS symptoms and likely keep exploring other interventions. Recently, scientific investigation and clinical research have begun to examine interventions targeting the gut microbiome. It has been hypothesized that microbiome dysbiosis leads to symptoms and dysbiosis may be corrected through probiotics, prebiotics, and synbiotics [5]. Probiotics are defined as live microorganisms that confer health benefits on the host when administered in adequate amounts [6]. Prebiotics are substrates that are selectively utilized by host microorganisms to confer health benefits and synbiotics are mixtures of live microorganisms and substrates that are selectively utilized by host microorganisms to confer health benefits [7, 8]. Contrary to popular belief, synbiotics are not simply mixtures of probiotics and prebiotics. In other words, the live microorganisms and substrates comprising synbiotics are not necessarily standalone probiotics and prebiotics. The International Scientific Association for Probiotics and Prebiotics categorizes synbiotics according to the relationship between the live microorganisms and the substrates. Synergistic synbiotics contain substrates that are selectively utilized by the co-administered or allochthonous microorganisms [8]. On the other hand, complementary synbiotics contain substrates that are selectively utilized by the resident or autochthonous microorganisms [8]. This narrative review summarizes randomized–controlled trials regarding probiotics, prebiotics, and synbiotics for the management of IBS.

Although the gut–brain (GB) axis has been extensively published as the framework for IBS, the gut–brain–microbiome (GBM) axis has recently moved into the spotlight. The bidirectional interactions underlying the GBM axis depend on interoception, which involves the posterior insula sensing information regarding the internal state of the body and then the anterior insula processing, and integrating this information with cognitive and emotional processes [9]. Interoception gives rise to homeostatic feedback loops, such as that within the enteric nervous system. This feedback loop regulates gastrointestinal motility, blood flow, and secretion during the physiological state and is modulated by the autonomic nervous system during threats to the physiological state [9]. The GBM axis proposes that the gut microbiome participates in this feedback loop through signaling molecules that act locally on enteric neurons, vagal afferents, and sympathetic afferents, as well as distantly acting on the CNS [9]. The main types of signaling molecules are food-derived molecules, host-derived molecules, and microbe-derived molecules.

**Figure 1:**
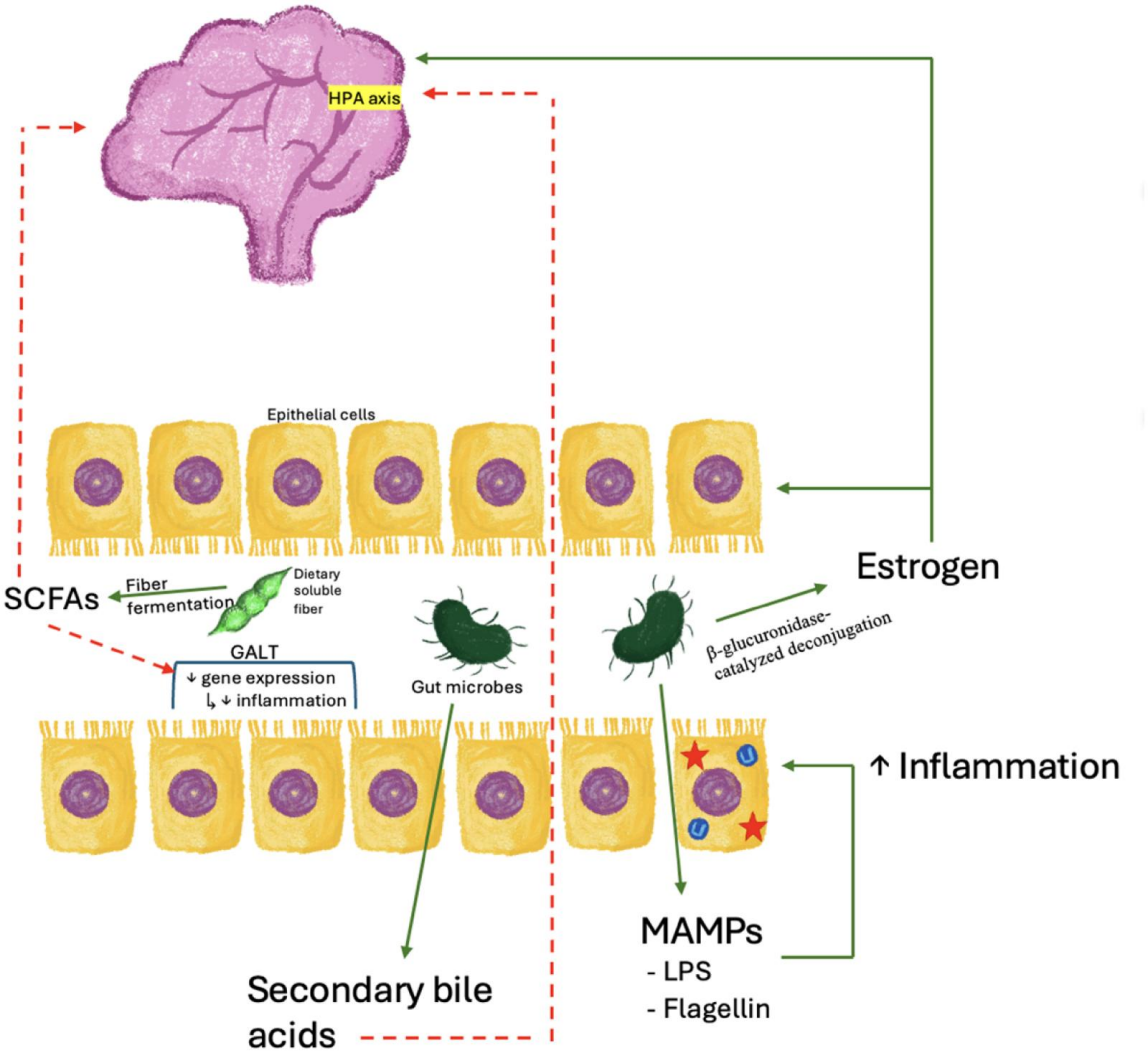
GBM and impact of food-derived molecules, host-derived molecules, and microbe-derived molecule. The production of SCFAs affect gene expression and, further downstream, decrease inflammation; host secondary bile acids accomplish a similar goal through a different mechanism and directly suppress the HPA axis. MAMPs oppose the actions of these molecules and increase inflammation in the gut epithelium.

Food-derived molecules include short-chain fatty acids (SCFAs), which are exclusively produced by the gut microbiome during dietary fiber fermentation [9]. SCFAs suppress afferents projecting to the brainstem and downregulate gene expression in the gut-associated lymphoid tissue, desensitizing homeostatic feedback loops and reducing gastrointestinal inflammation [9]. Host-derived molecules include secondary bile acids and estrogen. Secondary bile acids, which are generated by certain gut microbiome bacteria through primary bile acid transformation, regulate glucose homeostasis and suppress the hypothalamic–pituitary–adrenal (HPA) axis [9]. Likewise, estrogen is generated by certain gut microbiome bacteria through β-glucuronidase-catalysed deconjugation [9]. Previous studies have found that decreased estrogen levels in postmenopausal women contribute to increased IBS symptoms [9]. Microbe-derived molecules, or microbe-associated molecular patterns (MAMPs), include lipopolysaccharide and flagellin [9]. MAMPs stimulate toll-like receptors (TLRs) and recruit macrophages, neutrophils, and dendritic cells [9]. These gut-associated immune cells secrete pro-inflammatory cytokines that activate vagal afferents as well as microglia, disturbing homeostatic feedback loops [9]. The relationships between these various molecules and the GBM axis are depicted in Fig. 1.

Physical barriers block these signaling molecules, policing their role in the GBM axis. The intestinal barrier consists of mucus overlying epithelial cells that are connected by tight junctions and it separates the gut microbiome from the gut-associated immune system [9]. The mucus accommodates microbes by supporting biofilm formation and providing glycan nutrients [9]. At the same time, the mucus protects the epithelial cells via antimicrobial peptides and secretory immunoglobulin A [9]. The gut microbiota strengthens the intestinal barrier by producing SCFAs that maintain tight junctions [9]. Under the circumstances of dietary fiber deprivation or chronic stress, however, the gut microbiota weakens the intestinal barrier by overconsuming glycan nutrients [9]. Without the mucus protecting the epithelial cells, MAMPs stimulate TLRs, leading to the recruitment of gut-associated immune cells, secretion of pro-inflammatory cytokines, loosening of tight junctions, and perpetuation of metabolic endotoxemia [9]. Representing another physical barrier in the GBM axis, the blood–brain barrier (BBB) separates blood from the cerebrospinal fluid [9]. The gut microbiome reinforces the BBB by upregulating the gene expression of tight-junction proteins [9].

## Probiotics, prebiotics, and synbiotics for the management of IBS

Recent research emphasizes the possible relevance of the GBM axis in IBS. The present review thus focuses on exploring randomized, placebo-controlled, double-blind trials for interventions targeted at the GBM axis for IBS. Relevant studies were identified through PubMed. The literature search focused on trials involving adults diagnosed with IBS according to Rome III or IV diagnostic criteria who received treatments consisting of probiotics, prebiotics, or synbiotics. Studies examining gastrointestinal symptoms, global symptoms, or quality of life (QOL) were evaluated. The trials identified were summarized by subjects (number of participants, IBS diagnosis, Rome criteria), interventions (probiotic strains/prebiotic compounds/synbiotic mixture, daily dose, treatment period length, follow-up/washout period length), and outcomes (symptom measurement scale, *P* value).

Preclinical animal studies have sought to identify these changes in animal models as well. Martín *et al*. were able to establish that a *Bifidobacterium* strain was able to normalize tight junctions and increase colonic goblet cell populations to restore intestinal barrier function in mice with low-grade gut inflammation [10]. *Lactobacillus* species showed similar efficacy in increasing mucus production and decreasing gut permeability [11]. In mice models of post-infectious IBS, *Bifidobacterium* and *Lactobacillus* (but not *Streptococcus*) decreased visceral hypersensitivity (represented by the abdominal withdrawal reflex score) and reduced gut permeability [12]. A prebiotic combination of inulin and oligofructose was also efficacious in animal models and caused a shift in the microbiota towards *Bifidobacteria* and SCFA production, which can be assumed to lead to improved gut permeability as a result [13].

## Brief overview of treatment approaches for IBS

Interventions to treat IBS are varied and work through multiple mechanisms. Interventions target one or more components of the GB axis. Dietary treatments are typically first-line therapy. The low-FODMAP (fermentable oligosaccharides, disaccharides, monosaccharides, and polyols) diet aims to reduce gastrointestinal water secretion and colonic fermentation to decrease luminal distention. By limiting the fermentable substrates available to intestinal bacteria, this diet also modulates the microbiome, highlighting potential interactions between this dietary change and biotic therapies. Other dietary interventions include the British Dietetic Association/National Institute for Health and Care Excellence (NICE) diet. This intervention recommends eating small regular meals; avoiding late-night meals; reducing coffee, tea, and alcohol; limiting the intake of rich or fatty foods; and encouraging fiber intake [14]. The ACG also recommends soluble fiber for the treatment of IBS-C symptoms [15]. Soluble fiber increases gastrointestinal water secretion and decreases colonic fermentation, which may improve the viscosity and frequency of defecation. Laxatives such as polyethylene glycol are also widely used for IBS-C and work similarly to fiber in alleviating constipation. Peppermint oil can also be a helpful dietary supplement for people with IBS; the proposed mechanism posits that l-menthol encourages smooth-muscle relaxation [15].

Pharmaceuticals such as loperamide (a μ-opioid agonist) and alosetron (a 5-HT3 antagonist) may be useful for diarrheal IBS subtypes, as they slow down intestinal clearance [16]. The ACG also recommends chloride channel activators, such as lubiprostone, as well as guanylate cyclase-C agonists, such as linaclotide and plecanatide, which promote motility, for the treatment of IBS-C symptoms [15].

Antidepressants, including selective serotonin reuptake inhibitors (SSRIs) and tricyclic antidepressants (TCAs), are also used in the treatment of IBS. They have proven beneficial in reducing abdominal pain, slowing gastrointestinal transit, and reducing concomitant psychological stress.

Therapies targeting the microbiome have also become part of IBS treatment algorithms. Fecal microbiota transplantation (FMT) is a procedure that involves the infusion of feces from one or more healthy donors into the intestinal tract of a patient to enhance the recipient’s gut-microbiota diversity. El-Salhy *et al*. conducted clinical trials on 125 patients and determined that, 3 years after an FMT in patients with IBS, the response rates were 64.9% and 71.8% in the groups treated with 30 and 60 grams of feces, respectively, in comparison with the placebo group, who had a response rate of 27% [17].

Physical exercise is another approach for managing the symptoms of IBS. Zhou *et al*. conducted a systematic review of randomized–controlled trials and determined that, after 12 weeks of moderate-intensity exercise, a significant reduction in abdominal pain, abdominal distension, as well as anxiety and depression, was observed [18]. In addition, scores via the Irritable Bowel Syndrome-Severity Scoring System (IBS-SSS), which is used to measure the severity and frequency of abdominal pain, distension, and overall interference with everyday life, were significantly lower than those of the control groups. The ACG recommends physical exercise and emphasizes that it is particularly helpful in alleviating constipation over abdominal pain or quality of life, but that this recommendation is weak due to the low quality of evidence at this time [19].

Antispasmodics such as hyoscine butyl bromide are commonly used to relieve symptoms of IBS, including abdominal pain, cramping, and impaired motility, by increasing the colonic transit time, improving stool consistency, and decreasing the frequency of bowel movements [20]. However, antispasmodics are not recommended by the ACG as a treatment option for IBS in the USA due to limited supporting data for agents approved in the USA. The ACG notes the caveat that antispasmodics may be included in treatment algorithms internationally, as alternative antispasmodics available, such as alverine citrate, are better supported by clinical evidence [21].

**Figure 2:**
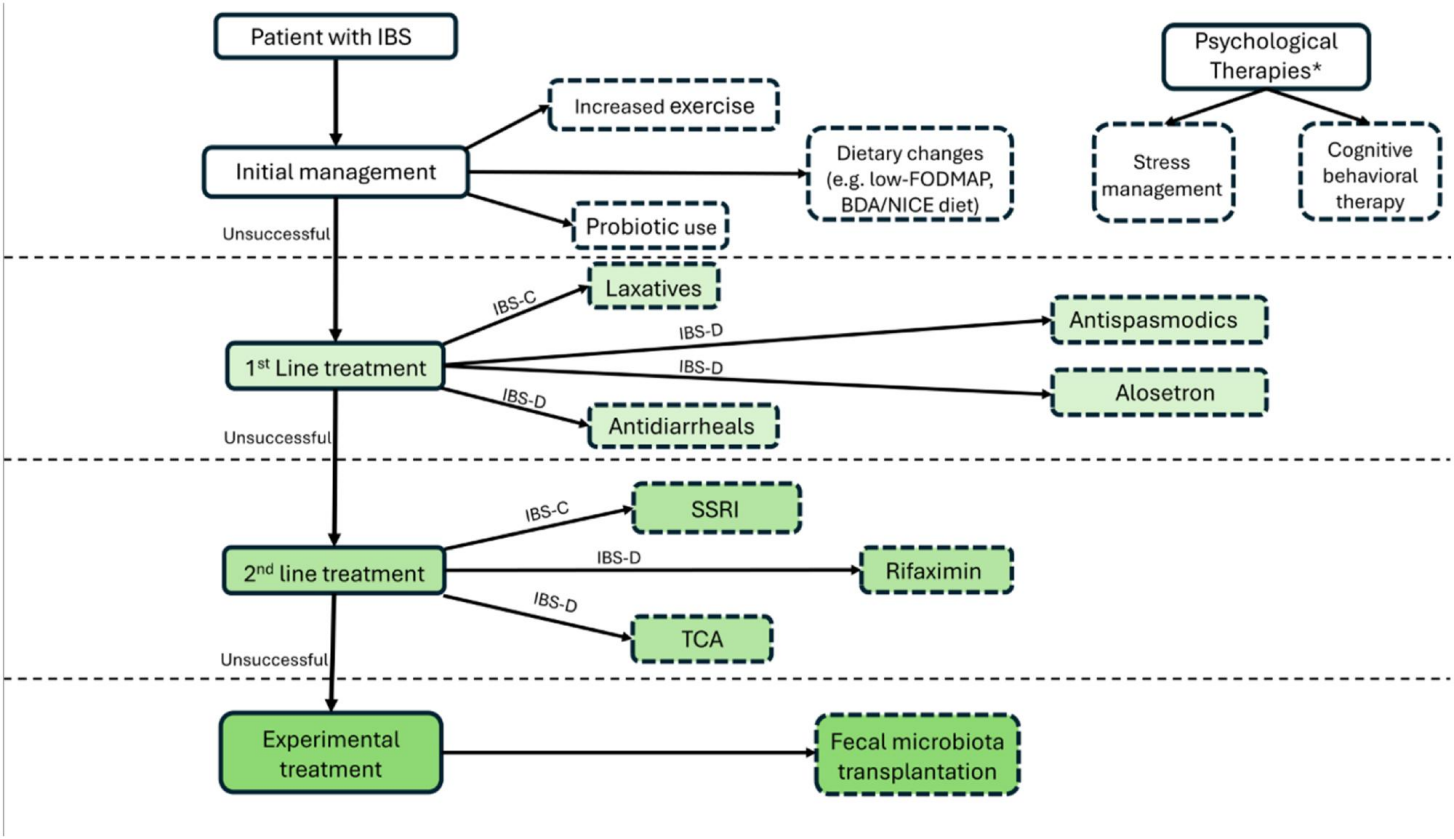
Schematic of overall approaches for the treatment of IBS. Outline of the potential steps taken to treat patients presenting with IBS from lifestyle changes to experimental treatments. FODMAP = fermentable oligosaccharides, disaccharides, monosaccharides, and polyols. * indicates treatment that may be started at any time point.

Finally, the ACG recommends rifaximin for the treatment of IBS-D symptoms [15]. This low bioavailable antibiotic presumably rebalances the gut microbiome with little risk of systemic toxicity. The direct mechanism of action of rifaximin is not fully understood; however, evidence points towards its anti-inflammatory properties on the gastrointestinal (GI) mucosa. A schematic for the overall approaches for the treatment of IBS is shown in Fig. 2.

## Clinical trials of probiotics, prebiotics, and synbiotics for the management of IBS

The outcomes of 31 relevant trials are presented: 22 trials regarding probiotics (Table 1), 3 trials regarding prebiotics (Table 2), and 6 trials regarding synbiotics (Table 3).

**Table 1:** Randomized, placebo-controlled, double-blind trials regarding probiotics for the treatment of IBS.

Strain	Subjects	Interventions	Outcomes
*Bacillus coagulans*	40 adults [31] IBS according to Rome IV criteria	*Bacillus coagulans* LBSC 6 billion CFUs/day for 8 weeks	Reduced symptom frequency according to DSFQ, especially abdominal-pain frequency (*P *< 0.0001) Reduced symptom severity according to IBS-SSS (*P* < 0.0001) Improved stool consistency according to BSFS (*P* = 0.0002)
	136 adults [32] IBS according to Rome III criteria	*Bacillus coagulans* Unique IS2 2 billion CFUs/day for 8 weeks Follow-up for 2 weeks	Reduced abdominal-pain severity (*P* < 0.0001) Increased frequency of CSBM (*P* < 0.0001) Reduced overall symptom severity (*P* < 0.0001) Caused a significant proportion of subjects to experience normal stool consistency according to BSFS (*P* < 0.0001) Caused a significant proportion of subjects to experience complete or considerable relief of global symptoms according to SGA and PGA (*P* < 0.0001)
*Lactobacillus*	80 adults [33] IBS according to Rome III criteria	2 strains (*Lactobacillus acidophilus* NCFM and *Lactobacillus helveticus* LAFTI L10) 10 billion CFUs/day for 8 weeks	Did not reduce abdominal pain (*P = *0.06), bloating (*P = *0.10), or rumbling (*P = *0.21) according to VAS Reduced flatulence according to VAS (*P = *0.03) Produced a significant composite response, defined as a combination of reduced abdominal pain, bloating, flatulence, and rumbling (*P = *0.04)
	50 adults [34] IBS (not IBS-C) according to Rome III criteria	3-strain Foodis *Lactobacillus* (*L. paracasei*, *L. salivarius*, and *L. plantarum*) 1 billion CFUs/day for 4 weeks	Caused a significant proportion of subjects to experience adequate relief of global symptoms according to SGA (*P = *0.009) Caused a significant proportion of subjects to experience ≥30% reduction in abdominal-pain severity according to VAS (*P = *0.048)
	214 adults [35] IBS according to Rome III criteria	*Lactobacillus plantarum* 299v 10 billion CFUs/day for 4 weeks Follow-up for 3 weeks	Reduced severity of abdominal pain (*P* < 0.05), bloating, and sense of incomplete evacuation (*P* < 0.05) Reduced frequency of abdominal pain (*P* < 0.05), bloating, sense of incomplete evacuation (*P* < 0.05), and stool frequency (*P* < 0.05)
	52 adults [36] IBS according to Rome III criteria	*Lactobacillus casei* subsp. *rhamnosus* LCR35 600 million CFUs/day for 4 weeks Follow-up for 2 weeks	Did not reduce symptom severity according to IBS-SSS (*P* = 0.8829)
*Bifidobacterium*	122 adults [37] IBS according to Rome III criteria	*Bifidobacterium bifidum* MIMBb75 1 billion CFUs/day for 4 weeks Washout for 2 weeks	Improved global symptoms according to SGA (*P* < 0.0001) Produced a significant composite response (*P* < 0.0001), defined as combination of reduced abdominal pain (*P* < 0.0001), bloating (*P* < 0.0001), and urgency (*P* = 0.0001) Did not improve stool frequency or sense of incomplete evacuation Improved bowel-habit satisfaction (*P* < 0.0001) Improved health-related quality of life according to SF-12, especially physical health (*P* = 0.0185) and mental health (*P* = 0.0083)
	443 adults [38] IBS according to Rome III criteria	Heat-inactivated *Bifidobacterium bifidum* HI-MIMBb75 1 billion CFUs/day for 8 weeks Washout for 2 weeks	Produced a composite response, defined as a combination of ≥30% reduction in abdominal-pain severity and adequate relief of global symptoms (*P* = 0.0007) Reduced symptom severity according to IBS-SSS (*P* = 0.0013), especially abdominal-pain frequency (*P* = 0.0080), bowel-habit satisfaction (*P* = 0.021), and interference with life (*P* = 0.012) Improved quality of life according to SF-12 (*P* = 0.038)
*Lactobacillus* and *Bifidobacterium*	330 adults [39] IBS according to Rome IV criteria	Arm 1 (*Lactobacillus acidophilus* DDS-1) Arm 2 (*Bifidobacterium animalis* subsp. *lactis* UAB1a-12) 10 billion CFUs/day for 6 weeks	Reduced abdominal-pain severity according to APS-NRS (Arms 1 and 2, *P* = 0.001) Reduced symptom severity according to IBS-SSS (Arms 1 and 2, *P* < 0.001) Caused a significant proportion of subjects to experience normal stool consistency according to BSFS (Arm 1, *P* = 0.002) (Arm 2, *P* = 0.022) Improved quality of life according to IBS-QoL (Arm 1, *P* = 0.016) Improved mental stress according to PSS (Arm 1, *P* = 0.023)
	251 adults [40] IBS according to Rome III criteria	Arm 1 (*Lactobacillus paracasei* HA-196) Arm 2 (*Bifidobacterium longum* R0175) 10 billion CFUs/day for 8 weeks	Did not reduce symptom severity according to IBS-SSS (Arm 1, *P* = 0.9632) (Arm 2, *P* = 0.0763) Did not improve stool consistency of IBS-C subjects according to BSFS (Arm 1, *P* = 0.0721) (Arm 2, *P* = 0.9805) Did not improve stool consistency of IBS-D subjects according to BSFS (Arm 1, *P* = 0.2896) (Arm 2, *P* = 0.5955) Did not improve general health according to SF-36 or depression and anxiety according to HADS
	25 adults [41] IBS according to Rome IV criteria	2-strain Zircombi (*Bifidobacterium longum* BB536 and *Lactobacillus rhamnosus* HN001 with vitamin B6) 5 billion CFUs/day for 30 days and crossover treatment for 30 days	Reduced symptom severity according to IBS-SSS (*P* < 0.0001) Improved abdominal pain, bloating, relief with defection, and interference with quality of life according to VAS (*P* < 0.0001)
	150 adults [42] IBS-C according to Rome III criteria	2-strain Arm 1 (*Lactobacillus acidophilus* PBS066 and *Lactobacillus reuteri* PBS072) 3-strain Arm 2 (*Lactobacillus plantarum* PBS067 and *Lactobacillus rhamnosus* LRH020; *Bifidobacterium animalis* subsp. *lactis* BL050) Arm 1 (10 billion CFUs/day) or Arm 2 (15 billion CFUs/day) for 60 days Follow-up for 30 days	Caused a significant proportion of subjects to experience ≥30% reduction in bloating, abdominal pain, constipation, abdominal cramps, and flatulence (Arms 1 and 2, *P* < 0.001) Improved health-related quality of life according to HR-QoL (Arms 1 and 2, *P* < 0.001)
	131 adults [43] IBS according to Rome III criteria	3 strains (*Lactobacillus paracasei* subsp. *paracasei* F19 and *Lactobacillus acidophilus* La5; *Bifidobacterium* Bb12) 52 billion CFUs/day for 6 months Follow-up for 6 months	Did not cause a significant proportion of subjects to experience adequate relief of abdominal pain (*P* = 0.18) Did not improve gastrointestinal symptom severity according to GSRS-IBS (*P* = 0.460) Did not improve quality of life according to IBS-QoL (*P* = 0.535)
*Lactobacillus*, *Bifidobacterium*, and *Streptococcus*	51 adults [44] IBS-D according to Rome III criteria	10 strains (*Bifidobacterium breve* BB010, *longum* BL020, *bifidum* BF030, *lactis* BL040; *Lactobacillus rhamnosus* LR110, *Lactobacillus paracasei* LPC100, *Lactobacillus acidophilus* LA120, *Lactobacillus casei* LC130, *Lactobacillus plantarum* LP140; *Streptococcus thermophilus* ST250) 5 million CFUs/day for 8 weeks	Reduced symptom severity according to IBS-SSS (*P* = 0.005), especially abdominal-pain severity (*P* = 0.015) and interference with life (*P* = 0.016) Improved global symptoms according to IBS-GIS (*P* = 0.003)
	49 adults [45] IBS according to Rome III criteria	6-strain LacClean Gold-S (*Bifidobacterium bifidum* KCTC 12 199BP, *Bifidobacterium lactis* KCTC 11 904BP, *Bifidobacterium longum* 12 200BP; *Lactobacillus acidophilus* KCTC 11 906BP and *Lactobacillus rhamnosus* KCTC 12 202BP; *Streptococcus thermophilus* KCTC 11 870BP) 10 billion CFUs/day for 4 weeks	Caused a significant proportion of subjects to experience adequate relief of global symptoms (*P* = 0.03) Did not improve abdominal pain (*P* = 0.07), abdominal discomfort (*P* = 0.35), bloating (*P* = 0.86), or stool frequency (*P* = 0.19) Did not improve stool consistency according to BSFS (*P* = 0.68)
	108 adults [46] IBS according to Rome III criteria	4-strain Probio-Tec Quatro-cap-4 (*Bifidobacterium animalis* subsp. *lactis* BB-12; *Lactobacillus acidophilus* LA-5 and *Lactobacillus delbrueckii* subsp. *bulgaricus* LBY-27; *Streptococcus thermophilus* STY-31) 8 billion CFUs/day for 4 weeks Follow-up for 4 weeks	Improved abdominal pain (*P* < 0.01) and bloating (*P* = 0.03) according to VAS Did not cause a significant proportion of subjects to experience reduced sense of incomplete evacuation (*P* = 0.25) Caused a significant proportion of subjects to experience adequate relief of symptoms (*P* < 0.01)
	179 adults [47] IBS-C or IBS-M according to Rome III criteria	4 strains (*Bifidobacterium lactis* I-2494; *Streptococcus thermophilus* CNCM I-1630; *Lactobacillus delbrueckii* subsp. *bulgaricus* CNCM I-1632 and *Lactobacillus delbrueckii* subsp. *bulgaricus* CNCM I-1519) 27 billion CFUs/day for 12 weeks	Caused a significant proportion of subjects to experience adequate relief of global symptoms according to SGA (*P* = 0.004) Reduced symptom severity according to IBS-SSS (*P* = 0.028) Reduced symptom severity according to Birmingham IBS Symptom Scale (*P* < 0.022), especially constipation (*P* = 0.011) Improved abdominal pain (*P* = 0.016) and bloating (*P* = 0.006) Did not improve quality of life according to IBS-QoL (*P* = 0.810)
*Lactobacillus*, *Bifidobacterium*, *Lactococcus*, and *Streptococcus*	360 adults [48] IBS-D according to Rome III criteria	14-strain Bio-Kult (*Bacillus subtilis* PXN 21; *Bifidobacterium bifidum* PXN 23, *Bifidobacterium breve* PXN 25, *Bifidobacterium infantis* PXN 27, *Bifidobacterium longum* PXN 30; *Lactobacillus acidophilus* PXN 35, *Lactobacillus delbrueckii* subsp. *bulgaricus* PXN39, *Lactobacillus casei* PXN 37, *Lactobacillus plantarum* PXN 47, *Lactobacillus rhamnosus* PXN 54, *Lactobacillus helveticus* PXN 45, *Lactobacillus salivarius* PXN 57; *Lactococcus lactis* PXN 63, *Streptococcus thermophilus* PXN 66) 8 billion CFUs/day for 4 months Follow-up for 1 month	Reduced symptom severity according to IBS-SSS (*P* < 0.001), especially abdominal-pain severity (*P* < 0.001) Improved quality of life according to IBS-QoL (*P* < 0.001)
*Lactobacillus* and *Enterococcus*	186 adults [49] IBS according to Rome III criteria	4-strain Symprove (*Lactobacillus rhamnosus* NCIMB 30174, *Lactobacillus plantarum* NCIMB 30173, *Lactobacillus acidophilus* NCIMB 30175; *Enterococcus faecium* NCIMB 30176) 1 mL/kg/day (with 50 mL = 10 billion CFUs) for 12 weeks Follow-up for 4 weeks	Reduced symptom severity according to IBS-SSS (*P* = 0.01), especially abdominal-pain severity (*P* = 0.03) and bowel-habit satisfaction (*P* = 0.01) Did not improve quality of life according to IBS-QoL (*P* = 0.47)
*Lactobacillus* and *Pediococcus*	84 adults [50] IBS-D according to Rome III criteria	3-strain I.31 (*Lactobacillus plantarum* CECT7484, *Lactobacillus plantarum* CECT7485, *Pediococcus acidilactici* CECT7483) Arm 1 (10–30 billion CFUs/day) or Arm 2 (3–6 billion CFUs/day) for 6 weeks	Improved quality of life according to IBS-QoL (Arm 1, *P* = 0.041) (Arm 2, *P* = 0.023), especially mental health (*P* = 0.030) Improved gut-related anxiety according to VSI (Arm 1, *P* = 0.033) (Arm 2, *P* = 0.015) Did not cause a significant proportion of subjects to experience complete or considerable relief of symptoms (Arms 1 and 2, *P* = 0.467)
*Clostridium butyricum*	200 adults [51] IBS-D according to Rome III criteria	135 million CFUs/day for 4 weeks	Reduced symptom severity according to IBS-SSS (*P* = 0.038), especially bowel-habit satisfaction (*P* = 0.014) and interference with life (*P* = 0.018) Improved quality of life according to IBS-QoL (*P* = 0.032), especially interference with activity (*P* = 0.003) and health worry (*P* < 0.001) Did not improve stool consistency according to BSFS (*P* = 0.259) Improved stool frequency (*P* = 0.035)
*Saccharomyces cerevisiae* CNCM I-3856	179 adults [52] IBS according to Rome III criteria	8 billion CFUs/day for 8 weeks Washout for 3 weeks	Did not reduce abdominal-pain severity between groups (*P* = 0.13) Caused a higher proportion of subjects receiving probiotic over placebo to experience ≥50% reduction in abdominal-pain severity (*P* = 0.04) Did not improve bloating, bowel-movement difficulty, or stool consistency Did not improve stool consistency according to BSFS

IBS-GIS = IBS Global Improvement Scale, DSFQ = Digestive Symptom Frequency Questionnaire, BSFS = Bristol Stool Form Scale, VAS = Visual Analogue Scale, APS-NRS = Abdominal Pain Severity Numeric Rating Scale, IBS-QoL = IBS Quality of Life, PSS = Perceived Stress Scale, SF-36 = Short Form 36 Health Survey, HADS = Hospital Anxiety and Depression Scale, SF-12 = Short Form 12 Health Survey, SGA = Subject’s Global Assessment, CSBM = Complete Spontaneous Bowel Movements, PGA = Physician’s Global Assessment, HR-QoL = Health-Related Quality of Life, VSI = Visceral Sensitivity Index, GSRS-IBS = Gastrointestinal Symptom Rating Scale for IBS.

**Table 2:** Randomized, placebo-controlled, double-blind trials regarding prebiotics for the treatment of IBS.

Subjects	Intervention	Results
61 adults [53] IBS according to Rome IV criteria	2′-*O*-fucosyllactose and lacto-*N*-neotetraose Arm 1 (5 g/day) or Arm 2 (10 g/day) for 4 weeks Follow-up for 4 weeks	Did not reduce gastrointestinal symptom severity according to GSRS-IBS or symptom severity according to IBS-SSS Did not improve anxiety or depression
60 adults [54] IBS-D according to Rome III criteria	Gelsectan (xyloglucan, PPT from grape-seed extract, and XOS) Treatment for 28 days and crossover treatment for 28 days Follow-up for 60 days	Caused a significant proportion of subjects to experience normal stool consistency according to BSFS (*P* = 0.0001) Caused a significant proportion of subjects to experience reduced abdominal-pain severity (*P* = 0.027) and bloating severity (*P* = 0.041)
79 adults [55] IBS according to Rome III criteria	Short-chain fructooligosaccharides (37% GF2, 53% GF3, 10% GF4) 5 g/day for 4 weeks	Did not reduce symptom severity according to IBS-SSS (*P* = 0.721)

GSRS-IBS = Gastrointestinal Symptom Rating Scale for IBS, BSFS = Bristol Stool Form Scale.

**Table 3:** Randomized, placebo-controlled, double-blind trials regarding synbiotics for the treatment of IBS.

Strain	Subjects	Interventions	Outcomes
*Lactobacillus*	67 adults [56] IBS according to Rome IV criteria	*Lactobacillus paracasei* DKGF1 and *Opuntia humifusa* (eastern prickly pear cactus) 100 billion CFUs/day and 0.2 g/day for 4 weeks	Caused a significant proportion of subjects to experience adequate relief of global symptoms according to SGA (*P* = 0.017) Caused a significant proportion of subjects to experience improved abdominal pain (*P* = 0.038) and psychological well-being (*P* = 0.004) according to VAS Did not cause a significant proportion of subjects to experience improved flatulence (*P* = 0.880) or bloating (*P* = 0.880) according to VAS Caused a significant proportion of subjects with IBS-C or IBS-D to experience ≥30% reduction in abdominal-pain severity according to VAS and improvement in stool frequency and consistency according to BSFS (*P* = 0.04)
*Lactobacillus* and *Bifidobacteria*	80 adults [57] IBS-D according to Rome III criteria	5 strains (3 *Bifidobacteria*, 2 *Lactobacilli*) and short-chain fructooligosaccharides (44% GF2, 46% GF3, 10% GF4) 10 billion CFUs/day and 947 mg/day for 8 weeks	Reduced symptom severity according to IBS-SSS (*P* = 0.042), especially abdominal-distention severity (*P* = 0.028) Improved global symptoms according to IBS-GIS (*P* = 0.043)
	30 adults [58] IBS according to Rome III criteria	8-strain Ultra-Probiotics-500 (*Lactobacilli rhamnosus*, *Lactobacilli acidophilus*, *Lactobacilli casei*, *Lactobacilli bulgaricus*, *Lactobacilli plantarum*, *Lactobacilli salivarius*; *Bifidobacteria bifidum*, *Bifidobacteria longum*) and fructooligosaccharides, *Ulmus davidiana* (slippery-elm bark), *Geum urbanum* (herb bennet), inulin Arm 1 (20 billion CFUs/day and 870 mg/day) or Arm 2 (10 billion CFUs/day and 435 mg/day) for 8 weeks	Improved abdominal pain (Arm 1, *P* = 0.002), bloating (Arm 1, *P* = 0.003), stool frequency (Arm 1, *P* = 0.002), and fatigue (Arm 1, *P* = 0.013) according to VAS Reduced fatigue severity according to MFI (Arm 1, *P* = 0.037)
*Bacillus coagulans*	85 adults [59] IBS according to Rome III criteria	Lactol (*Bacillus coagulans* and FOS) 450 million CFUs/day and 300 mg/day for 12 weeks Follow-up for 9 months	Reduced frequency of abdominal pain (*P* < 0.001) and loose stools (*P* < 0.001) according to Rome III questionnaire Did not reduce frequency of hard stools according to Rome III questionnaire (*P* = 0.561)
*Saccharomyces*	72 adults [60] IBS-D according to Rome III criteria	*Saccharomyces boulardii* and ispaghula husk 750 mg/day and “spoonful”/day for 6 weeks	Did not improve stool frequency (*P* = 0.200), urgency (*P* = 0.710), straining (*P* = 0.063), sense of incomplete evacuation (*P* = 0.056), stool consistency (*P* = 0.076), abdominal pain (*P* = 0.005), bloating (*P* = 0.421), and passage of mucus (*P* = 0.894) Improved quality of life according to IBS-QoL (*P* = 0.002), especially food avoidance (*P* = 0.030) and body image (*P* = 0.024)
*Lactobacillus*, *Bifidobacteria*, and *Streptococcus*	64 adults [61] IBS according to Rome III criteria	9-strain Probinul (*Lactobacillus plantarum*, *Lactobacillus casei* subsp. *rhamnosus*, *Lactobacillus gasseri*, *Lactobacillus acidophilus*, *Lactobacillus salivarius*, *Lactobacillus sporogenes*; *Bifidobacterium infantis*, *Bifidobacterium longum*, *Streptococcus thermophilus*) and inulin 38 billion CFUs/day and 4.4 g/day for 4 weeks	Did not cause a significant proportion of subjects to experience adequate relief of bloating (*P* = 0.21) or flatulence (*P* = 0.45) Improved flatulence according to VAS (*P* = 0.038) Did not improve bloating, abdominal pain, or urgency according to VAS

SGA = Subject’s Global Assessment, VAS = Visual Analogue Scale, BSFS = Bristol Stool Form Scale, IBS-GIS = IBS Global Improvement Scale, MFI = Multidimensional Fatigue Inventory, IBS-QoL = IBS Quality of Life.

Randomized, placebo-controlled trials on probiotics in IBS (Table 1) have shown generally positive results, though the choice of bacteria used seemed to have an impact on the clinical improvement. Both trials that used *B. coagulans* saw reduced severity of symptoms, reduced abdominal pain, and improved stool consistency. The results with *Lactobacillus* strains were more mixed. *L. acidophilus* NCFM and *L. helveticus* LAFTI L10 alone did not reduce abdominal pain, but a combination of *L. paracasei*, *L. salivarius*, and *L. plantarum* reduced abdominal pain and global symptoms. *L. plantarum* alone also showed a positive effect, reducing abdominal pain and decreasing stool frequency. *L. casei* subsp. *rhamnosus* did not reduce symptom severity. *Bifidobacterium* was generally efficacious. *B. bifidum* improved global symptoms and health-related quality of life, although it did not improve stool frequency. Heat-inactivated *B. bifidum* similarly reduced symptom severity and improved quality of life, suggesting that heat-inactive probiotics are not necessarily less effective when used for IBS. *B. animalis* subsp. *lactis* reduced abdominal pain and symptom severity. *B. longum*, however, neither reduced symptoms, improved stool consistency, nor improved quality of life. In trials, a combination of *Lactobacillus* and *Bifidobacterium* seemed to be more efficacious in reducing symptoms than *Lactobacillus* alone. *B. longum* and *L. rhamnosus* together reduced symptom severity and improved abdominal pain. *L. plantarum* and *L. rhamnosus* with *B. animalis* subsp. *lactis* reduced abdominal pain, bloating, constipation, and flatulence while improving quality of life. *L. paracasei* and *L. acidophilus* with *Bifidobacterium bb12*, however, did not improve symptoms. A 10-strain combination of various *Bifidobacterium*, *Lactobacillus*, and *S. thermophilus* did reduce symptom severity and improved global symptoms, suggesting that an increased diversity of strain may have an enhanced positive impact. Another combination of *Lactobacillus*, *Bifidobacterium*, and *Streptococcus* with six strains improved global symptoms but not abdominal pain, stool frequency, or stool consistency when looked at in isolation. Two other trials looked at four strain combinations of *Lactobacillus*, *Bifidobacterium*, and *Streptococcus*, and the results of both did show improved abdominal pain and the relief of symptoms in general. A 14-strain combination of *Lactobacillus*, *Bifidobacterium*, *Lactococcus*, and *Streptococcus* showed very positive results in reducing symptom severity and improving quality of life. A four-strain *Lactobacillus–Enterococcus* combination improved symptom severity, particularly abdominal pain. A three-strain *Lactobacillus*–*Pediococcus* combination improved quality of life, particularly mental health. *Clostridium butyricum* also reduced symptom severity and improved quality of life. *Saccharomyces cerevisiae* did not alter stool consistency but did cause a higher proportion of participants to experience a reduction in abdominal pain. For many of these trials, doses used were in the range of billions of colony forming units (CFUs)/day for weeks to months—even as high as 52 billion CFUs per day for 6 months.

There are much fewer trials investigating the effects of prebiotics. Results were less convincing for the three trials that were relevant to this review than those of probiotics. Smaller study sample sizes may have also contributed to these results. A combination of 2′-*O*-fucosyllactose and lacto-*N*-neotetraose, as well as short-chain fructooligosaccharides (FOS), was not effective in improving GI symptoms, and a blend of 2′-*O*-fucosyllactose and lacto-*N*-neotetraose did not improve anxiety or depression. Gelsectan, however, was found to improve abdominal-pain severity and stool consistency.

Six trials are included regarding synbiotics. *L. paracasei* with prickly pear improved global symptoms, abdominal pain, and stool frequency but not bloating or flatulence. Multi-strain combinations of *Lactobacillus* and *Bifidobacterium* with FOS reduced the severity of IBS symptoms and improved global symptoms. An eight-strain combination of *Lactobacilli* and *Bifidobacteria* with FOS improved multiple outcomes, including abdominal pain, bloating, stool frequency, and fatigue. *B. coagulans* with FOS reduced abdominal pain and loose stools but not hard stools. *S. boulardii* with ispaghula husk did not improve most IBS symptoms but did enhance quality of life. A nine-strain combination of *Lactobacillus*–*Bifidobacterium*–*S. thermophilus* improved flatulence but not bloating or abdominal pain. All six trials employed a synbiotic that relieved at least one IBS symptom. The selection of synbiotic should likely be targeted at the most debilitating symptoms that the patient has.

It will be informative to know the way in which each of these biotics influences symptom relief for patients with various IBS subtypes. Not all of these trials include information about IBS subtypes. For IBS-D patients, *Lactobacillus* alone and in combination with *Bifidobacterium* reduced global symptoms and abdominal pain. *Lactobacillus* with either *Bifidobacterium–Lactococcus–Streptococcus* or with *Pediococcus* improved quality of life, though the *Lactobacillus–Bifidobacterium*–*Lactococcus*–*Streptococcus* combination reduced abdominal pain and the *Lactobacillus*–*Pediococcus* combination improved gut-related anxiety. *C. butyricum* is also beneficial in IBS-D, as it reduced symptom severity, improved QOL, and improved stool frequency, though it did not improve consistency. For IBS-C patients, *Lactobacillus*–*Bifidobacterium* combinations significantly reduced bloating, abdominal pain, constipation, abdominal cramps, and flatulence. This combination also improved quality of life. For IBS-C and IBS-M patients, a *Lactobacillus*–*Bifidobacterium*–*Streptococcus* combination provided the adequate relief of global symptoms, reduced symptom severity, improved abdominal pain, especially constipation, but did not improve quality of life. These data suggest that *Lactobacillus* or *Bifidobacterium* combinations may work particularly well for IBS-D, IBS-C, or IBS-M, though these data are limited in that many trials evaluated global symptom scores rather than subtype-specific outcomes. Overall, a two- or three-strain combination of *Lactobacilli*, *Bifidobacterium*, and *Streptococcus* was useful for IBS-C patients, while, for IBS-D patients, (i) a multiple-strain (10–14 strains) combination of *Bifidobacterium*, *Lactobacillus* and *Streptococcus*, (ii) a combination of xyloglucans and xylooligosaccharides, (iii) a five-strain combination of *Bifidobacteria* and *Lactobacilli* together with short-chain FOS, or (iv) a combination of *S. boulardii* and ispaghula husk was found to be useful.

## Discussion

Our literature search resulted in 22 trials regarding probiotics. Nineteen trials employed probiotics that relieved IBS symptoms, ranging from gastrointestinal symptoms to global symptoms to improving the quality of life. Most of the included trials showed that several probiotics, especially those containing *Lactobacillus* (in combination with other bacteria rather than used alone) and *Bifidobacterium*, provide clinically meaningful benefits. One trial utilizing a heat-inactivated *B. bifidum* preparation demonstrated symptomatic improvement, which may warrant further investigation into whether nonviable probiotic formulations could provide clinical benefit while mitigating safety concerns in immunocompromised or pediatric populations. As mentioned above, it was observed that clinical trials that used combinations of several strains had positive results. It will require further studies to differentiate whether more strains are better in terms of diversifying a microbiome in dysbiosis or *Lactobacillus* and *Bifidobacterium* led to the clinical improvement observed, especially as a few clinical trials showed that they were efficacious on their own. *B. coagulans* was also found to be efficacious in reducing symptoms.

Of the three trials that investigated prebiotics, no consensus was observed on whether there was significant clinical improvement after a trial period. Gelsectan alone improved symptoms. One difference between Gelsectan and the other prebiotics, though, is that Gelsectan is a combination of a prebiotic, namely xylooligosaccharide (XOS), with xyloglucan and pea protein and tannins (PPT), which are mucosal-protective. This suggests that mucosal-protective factors may confer benefit for IBS. Future studies may be needed to make this distinction. Only one prebiotic trial made mention of an IBS subtype. This trial documented the effect of Gelsectan on IBS-D and demonstrated normalized stool consistency, reduced abdominal pain, and reduced bloating severity. Six trials that investigated synbiotics of various species were included, all of which showed some form of symptomatic improvement. Two synbiotic trials made mention of an IBS subtype, namely IBS-D. A combination of *Lactobacillus*–*Bifidobacteria* reduced symptom severity, especially abdominal distention, and improved global symptoms, while *Saccharomyces* improved quality of life but did not improve frequency, consistency, abdominal pain, or bloating.

Comparing probiotics to synbiotics may also inform whether certain species need supportive elements to be able to confer benefits. Probiotic trials with *Lactobacillus* alone, such as *L. acidophilus*, *L. helveticus*, and *L. casei* subsp. *rhamnosus*, did not see significant clinical improvement, but *L. paracasei* with prickly pear was effective in decreasing symptoms, suggesting that the efficacy of some probiotic organisms could depend on environmental or nutritional factors within the gut. Synbiotics may enhance the colonization and functional impact on the intestinal microbiome. Experimental studies have also shown that the same combination of *L. paracasei* with prickly pear improved stool consistency and reduced inflammatory markers such as tumor necrosis factor-alpha (TNF-α) while enhancing intestinal barrier protein expression in an IBS animal model, suggesting a possible beneficial response to synbiotics [22]. However, large-scale meta-analyses evaluating microbiome-directed therapies in IBS show a comparative lower efficacy of synbiotics compared with probiotics. This is likely because of the heterogeneity in formulations and limited number of high-quality trials surrounding synbiotics [23]. However, these findings suggest that future research comparing individual probiotic strains with corresponding synbiotic formulations may help to clarify whether specific synbiotic formulations enhance the efficacy of IBS treatment.

**Figure 3:**
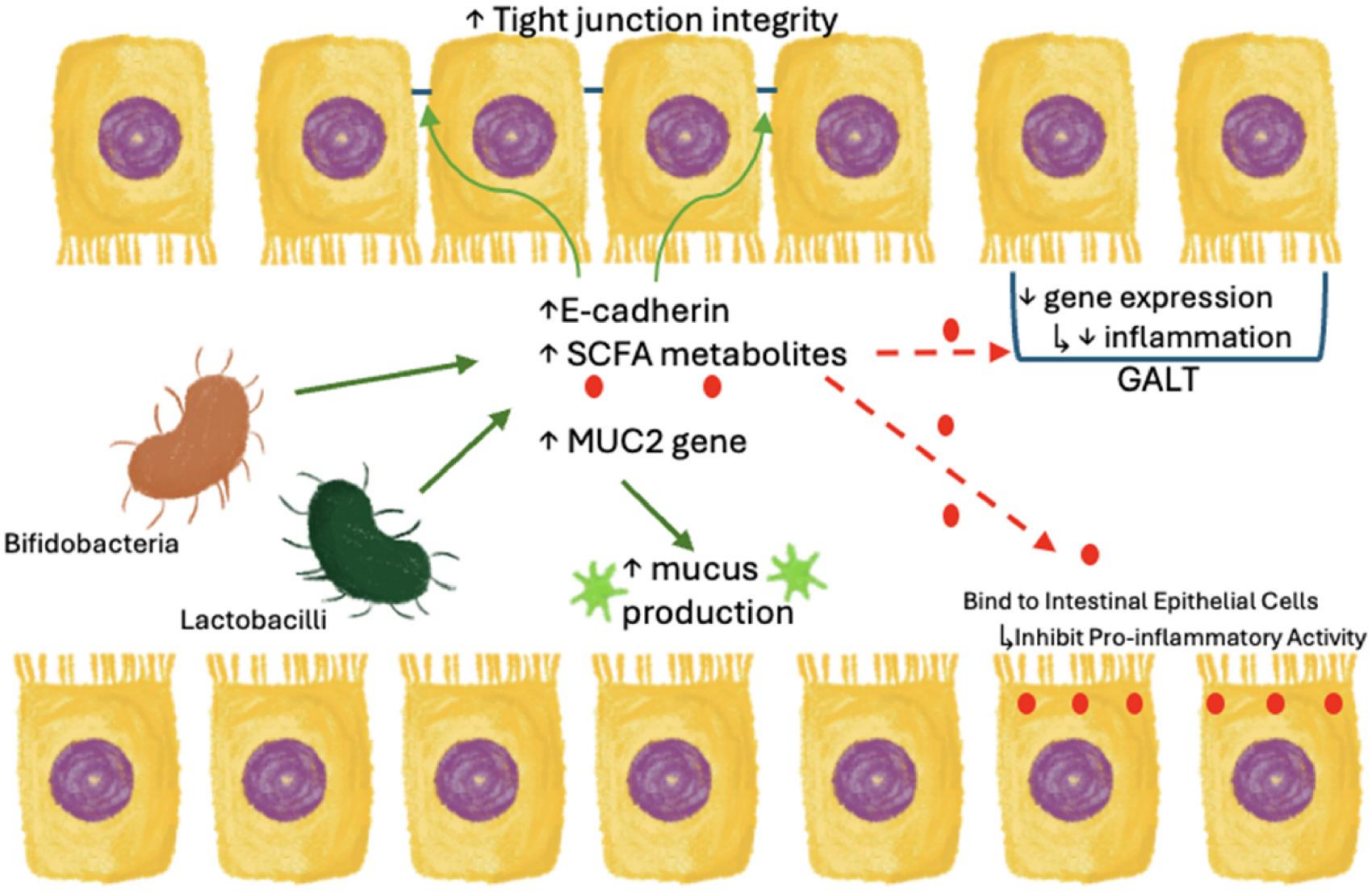
Impact of *Lactobacilli* and *Bifidobacteria* in maintenance of the gut epithelium. *Lactobacilli* and *Bifidobacteria* increase mucus production through stimulating MUC2 and strengthen the tight-junction barriers by producing E-cadherin. These bacteria also increase the production of SCFA metabolites, which directly bind epithelial cells to oppose inflammatory signals.

Two commonly used probiotics are *Lactobacilli* and *Bifidobacteria*. These organisms are rod-shaped, gram-positive bacteria that have, in previous studies, been shown to be involved in the intestinal barrier function. *Lactobacilli* and *Bifidobacteria* are thought to increase mucus production by upregulating the *MUC2* gene in colonic epithelial cells, as well as improving the tight-junction integrity of intestinal epithelial cells through an increase in the expression of E-cadherin and the production of SCFA metabolites [24, 25]. These metabolites also bind to intestinal epithelial cells, inhibiting pro-inflammatory activity (Fig. 3).

The most extensively studied prebiotics consist of fructans or FOS and galactans or galactooligosaccharides (GOS) [7]. These non-digestible oligosaccharides are preferentially metabolized by *Bifidobacteria*, which possess β-fructosidase and β-galactosidase enzymes that hydrolyse linkage bonds in FOS and GOS [7]. *Bifidobacteria* also favor carbohydrates with a degree of polymerization of between 4 and 30, which is characteristic of FOS and GOS [7]. This narrative review included three trials regarding prebiotics. Only one trial employed a prebiotic that relieved IBS symptoms, specifically gastrointestinal symptoms. The prebiotic product contained a mixture of compounds (xyloglucan, PPT from grape-seed extract, and XOS).

Probiotics have a long history of use in foods and dietary supplements, and are generally considered safe for human consumption. Adverse effects associated with probiotic administration are uncommon and typically mild, frequently including bloating or gas. Serious complications are rare and are largely reported in populations with significant underlying illnesses. Safety concerns associated with ingestion of probiotics and synbiotics include opportunities infections, particularly in immunocompromised patients with impaired intestinal barrier functions, though the incidence is extremely low and is estimated to occur in fewer than one case per million users for *Lactobacillus* species. Additional theoretical risks include the transfer of antibiotic resistance genes to the microbiota, deleterious metabolic activity, and immune modulation; however, these risks appear to be strain-specific and have not been shown to be widely demonstrated in clinical studies. Overall, the available evidence suggests that probiotics have a favorable safety profile in healthy individuals and those with FGIDs such as IBS [26].

Evidence examining combined dietary and probiotic therapy in IBS is limited, with only a small number of randomized–controlled trials directly assessing additive effects. These outcomes suggest that, while dietary interventions are the primary drivers of symptom improvement, probiotics may play a complementary but not consistently additive role. A randomized–controlled trial showed that a low-FODMAP diet significantly improved symptoms but reduced *Bifidobacterium* abundance, while probiotic supplementation restored microbial levels without enhancing symptom relief [27]. Similarly, another randomized–controlled trial found that adding probiotics to a low-FODMAP diet did not provide additional clinical benefit beyond the diet alone, despite overall symptom improvement [28]. Broader evidence from a systematic review and network meta-analysis supports this finding, concluding that, although both probiotics and dietary interventions are individually effective, their combined impact remains inconsistent and likely depends on strain specificity and patient heterogeneity [29]. Furthermore, research examining gut-microbiota responses to diet and probiotics indicates that the baseline microbial composition may influence treatment response, highlighting the complex interaction between dietary interventions and microbiome-targeted therapies [30]. Collectively, these studies suggest that probiotics function primarily as a microbiota-modulating adjunct to dietary therapy rather than enhancing symptom reduction, underscoring the need for more personalized combination approaches.

This review has certain limitations. First, the literature search was limited to PubMed, which may have excluded studies indexed elsewhere. Additionally, the outcome measures of the various studies were not identical, which limited direct comparison across studies. The review also included studies that used both Rome III and IV diagnostic criteria, potentially introducing some variability in the study population. Not all studies stratified patients into IBS subtype, which limits the ability to determine whether specific interventions are more effective for constipation- versus diarrheal-dominant IBS subtypes. Finally, many studies only looked at short treatment periods; therefore, the long-term efficacy remains unclear.

Future research should aim to compare probiotic and synbiotic formulations to further clarify whether the addition of substrates improves clinical benefit over probiotics alone. In addition, future trials should stratify patients by IBS subtype to determine whether specific strains or combinations are more efficacious for particular patient populations. Studies that perform pre-intervention microbiome testing to inform personalized therapeutic approaches should also be conducted to see whether the clinical benefit is greater than that when using a universal approach.

## Conclusion

IBS is increasingly thought of as a disorder of the GBM and interventions aimed at this axis are becoming increasingly popular. This review supports the use of probiotics and synbiotics—especially those containing *Lactobacillus* and *Bifidobacterium*—as agents that may modify the disordered GB interactions and consequent gastrointestinal symptoms that are responsible for FGIDs. Given the heterogeneity in treatment response observed across trials, future research should explore the potential for personalized approaches to microbiome-directed therapy in IBS. Variability in individual microbiome composition and host factors may influence treatment efficacy and identifying predictors of response could help guide more targeted interventions. Currently, evidence supporting the use of prebiotics is lacking. Future scientific investigation and clinical research should involve longitudinal trials to determine whether these interventions offer lasting health benefits. Future investigations are also needed to clarify the optimal bacterial strains, doses, and treatment durations.

## Authors’ contributions

S.P. and J.D. designed the study. Each author contributed to the drafting of the manuscript.
